# Narcea—an unknown, ancient cultivated rose variety from northern Spain

**DOI:** 10.1038/s41438-020-0266-8

**Published:** 2020-04-01

**Authors:** María-Carmen Martínez, José-Luis Santiago, Susana Boso, Pilar Gago, Inmaculada Álvarez-Acero, María-Estela De Vega, Miguel Martínez-Bartolomé, Rafael Álvarez-Nogal, Pilar Molíst, Matteo Caser, Valentina Scariot, Daniel Gómez-García

**Affiliations:** 1 Misión Biológica de Galicia (CSIC), Carballeira 8, Salcedo, 36143 Pontevedra Spain; 2 Instituto de Ciencia y Tecnología de Alimentos y Nutrición (CSIC) (Spain), C/José Antonio Novais 10, 28040 Madrid, Spain; 3 Departamento de Biología Molecular-Área de Biología Celular, Universidad de León, Campus de Vegazana s/n, 24071 León, Spain; 4 Grupo Neurolamb, Biología funcional y Ciencias de la Salud, Universidad de Vigo (Spain), 36310 As Lagoas-Marcosende, Spain; 5 Department of Agricultural, Forest and Food Sciences, University of Torino, Largo Paolo Braccini 2, 10095 Grugliasco, Turin Italy; 6 Instituto Pirenaico de Ecología (CSIC), Dpto. Conservación de Ecosistemas Naturales, Avda. Montaña S/N, Zaragoza, 50016 Zaragoza, Spain

**Keywords:** Plant domestication, Biodiversity

## Abstract

The present work reports the discovery and the complete characterisation of an ancient cultivated rose variety found growing in a private garden in the southwest of the Principality of Asturias (northern Spain). The variety is here given the name Narcea. The majority of roses currently cultivated belong to the so-called group of ‘Modern Roses’, all of which were obtained after 1867 via artificial crosses and improvement programmes. All are destined for ornamental use. Until the 19th century, the great majority of the many ancient cultivated roses in Europe were used in perfumery and cosmetics, or had medicinal uses. *Rosa damascena* and *Rosa centifollia* are still grown and used by the French and Bulgarian perfume industries. The Asturian Massif of the Cantabrian Mountain Range provides a natural habitat for some 75% of the wild members of the genus *Rosa*, but until now there was no evidence that this area was home to ancient cultivated roses. A complete botanical description is here provided for a discovered ancient rose. It is also characterised according to a series of sequence tagged microsatellite sites, and its agronomic features are reported. In addition, a histological description (optical and scanning electronic microscope studies) of the petals is offered, along with an analysis of the volatile compounds present in these organs as determined by solid phase microextraction and gas chromatography-mass spectroscopy. The results reveal the uniqueness of this ancient type of rose and suggest it may be of interest to the perfume industry.

## Introduction

This work describes an ancient cultivated rose variety found growing in a private garden in western Asturias (northern Spain). Many wild roses grow in this area, but it was never thought to be home to ancient cultivated roses. The histological, genetic and biochemical analysis of the examined material reveal the uniqueness of this ancient variety—here named *Narcea*—and suggest it may be of interest to the perfume industry.

The first thing to understand when discussing roses is that wild roses—those found growing along paths and tracks or in hedges lining meadows—are very different to cultivated roses. From a botanical standpoint, authors past and present coincide in describing the genus *Rosa* as one of the most complex and confusing, due to several factors including extensive hybridization and polyploidy. The genus *Rosa* can be divided into four subgenera^1^—*Hultemia* (Dumort) Focke, *Platyrhodon* (Hurts) Rheder, *Hesperhodos* Cockerell, and *Rosa* Focke—which together include the 150 or so rose species distributed around the northern hemisphere. The first three of these subgenera have been little involved in the appearance of current garden roses and the subgenus *Rosa* includes nearly all cultivated rose species^2^.

In their wild state, roses are generally simple plants with a single row of petals and numerous stamens^3^. However, cultivation led to the appreciation that rose stamens can sometimes transform into petals. This led to the appearance of cultivated roses with numerous petals and very few (or no) stamens. According to the degree of transformation they have undergone, cultivated roses are classified in terms of their number of petals as single, semi double, or double. In the first half of the 19th century, authors^3^ reported there to be over 100 species of cultivated rose within the genus *Rosa*, all from the northern hemisphere and with none from further south than 25°N. They also indicated that the cultivated roses of their time were rustic and abundant in southern Europe, and so numerous that many hybridisations are thought to have occurred (although in those days it was very hard to know the parent types involved or where they came from).

The great majority of the many ancient cultivated roses described in Europe in the 19th century^3–7^ have all but disappeared. All the latter authors, however, underscore *Rosa centifolia* (understood here to be a species, following the criteria of Koopman et al.^8^) as the rose *par excellence*. It was to this rose that was mostly grown in European gardens until species from China and India took its place (from 1867)^3^. Varieties of *R. centifoli*a also provided the majority of the raw material used in the important 19th century rose essence industry. It was unsure whether *R. centifolia* was a native of southern Europe, although by this time it had certainly become naturalised in that part of the world, and in the form of different varieties^3^.

Another ancient rose commonly mentioned by all 19th-century authors was *R. gallica* or the Provence rose. Authors^3^ described it as a different ‘race’ to *R. centifolia*, from which it was quite difficult to distinguish. The latter authors thought it unlikely that it arose as a hybrid of *R. centifolia* since it was so similar to it. However, the flowers of *R. centifolia* appear either alone or in groups of two or three, while the many varieties of *R. gallica* cultivated in the 19th century had flowers in groups of three or four, all growing from the same erect peduncle.

*R. damascena* was another ancient and much appreciated cultivated rose at the pass. This species, as well as *R. centifolia* and *R. gallica*, belongs to the subgenus Rosa (2× = 4n) that has a mainly European genetically background^8^. *R. damascena* is often treated as species in literature^8,9^. Morphologically it can be distinguishable by its long thorns, oblong fruit, the way its flowers gather into a corymb, and the ease with which it could be propagated from cuttings^3^.

*R. moschata* Herrm., an ancient rose with origin in the Persian region or in the Eastern Mediterranean, was widely cultivated in the North of Africa during the XVIII century and was probably already known in Roman times^10^. This rose has been cultivated for centuries to obtain its essential oil^10^ which is currently used in many cosmetic products. This rose grows naturalised in several localities of the North of the Iberian Peninsula.

Nowadays, the majority of cultivated roses belong to the “Modern Roses” group, and were obtained from 1867 (the year when the French nurseryman Jean-Baptiste André Guillot introduced ‘La France’, the legendary first Hybrid Tea Rose) onwards via artificial crossings and improvement programmes. The aim was to achieve ornamental plants for gardens, and cut flowers. These roses usually have elegant, aesthetically attractive flowers, and there are thousands of colours and forms—but all have little fragrance. The so-called “Ancient Roses”, all of which have now practically disappeared, are generally more rustic, more ungainly and less aesthetically pleasing. However, the roses of this group are those used in the making of perfume; the intense, exquisite fragrance produced by some varieties has never been reproduced in the laboratory.

Wild roses of different species are abundant in Spain, especially in the Aragonese Pyrenees^11^ and the west of Asturias (Supplementary Fig. S1)—the latter being home to perhaps some 75% of the species within the genus *Rosa*^12–14^. There is, however, but one dubious reference to the country ever having been home to a cultivated ancient rose. One *Rosal Castellano de flor muy doble* as belonging to *R. gallica* L. was mentioned, perhaps because of its Spanish name^15^. However, neither authors of this work^14^ nor anyone else mentions anything else about this variety, nor has a specimen confirming its existence been found.

For earlier authors, the characterisation and classification of cultivated roses was a complex business, due to their complex hybrid origin and polyploidy chromosomal series, but modern descriptive methods developed for the genus *Rosa*^11,16^, allow now all researchers to use the same methodology and terminology. This makes the description and comparison of varieties much easier. Classification and taxonomy of genus Rose is still complex due to the existence of hybridation and different polyploid levels^1^. Molecular techniques^17^ are also available, and these techniques are being applied to detect genetic relationships^8,9^ or to answer specific questions about varietal identity that botanical techniques alone cannot. Among the molecular markers available, sequenced tagged microsatellite sites (STMSs) are regarded as neutral markers that are more informative when characterizing germplasm collections thanks to their abundance in eukaryotic genomes, their high levels of polymorphism, Mendelian inheritance, co-dominant nature, and locus specificity^17,18^. STMSs markers have been widely employed in genetic diversity and mapping studies in rose^9,17,19–21^. Currently, thanks to next-generation sequencing, also single-nucleotide polymorphism (SNP) markers are available. They have lower information content per marker but automation makes possible to detect tens of thousands of markers simultaneously, resulting especially informative for allele dose determination^22^.

With regard to roses used in perfume production, agronomic characteristics such as the number of roses per bush, the size of flowers, the number of petals each flower has, and the weight of the petals, are also of interest (the essential oils used in perfumery are extracted from the petals). Knowledge of the histological characteristics of the petals is also important since it is in the petal tissues where these oils are stored. The content and concentration of the volatile compounds present in the essential oil determines the aromatic profile of a rose variety, and bears strongly on its importance to the perfume industry. Indeed, great varietal differences exist^23,24^ in terms of the characteristics, quality, intensity and persistence of a rose aroma. The final aroma is a product of the varying presence of citronelol, geraniol, nerol, linalool, phenylacetic alcohol and other compounds, which can be affected by the time when the petals are collected, soil type, climate, and cultivation practices^25–27^.

The aim of the present work was to botanically, genetically, histologically and biochemically characterise an unknown ancient, cultivated rose variety discovered in northern Spain, and to provide preliminary data of possible interest to the perfume industry. Should it generate such interest, it would be the first variety from Spain, and as far as we know only the third from Europe, to do so.

## Materials and methods

### Plant material and climate of the area where it was found

The examined plant material is from a very old, cultivated, domesticated type of rose found growing in the private garden of a house in the small village of Carballo (Concejo de Cangas del Narcea, in the Principality of Asturias, northern Spain), which nestles in the valley of the River Cibea. Several specimens of this type of rosebush may have been growing in this same garden before 1867 (and possibly before 1832) (personal communications from local inhabitants). Certainly, their long existence was known to several senior villagers. Their large roses with their many petals, notable colour and agreeable aroma, led to their being collected for use in the festival of Corpus Christi, the petals being thrown into the air as the religious procession passed by. Indeed, even if the flowering of the rose depends on temperature and humidity conditions, we could approximately predict flowering time thanks to the fact that the oral tradition had stablished that flowering occurred around the time of this festival (which falls on the ninth Sunday after the first spring full moon in the northern hemisphere). Today there are three such plants in the same garden; these are now being used to propagate more specimens.

Carballo lies in a mountainous area (with altitudes rising from 300 to 1700 m over just a few kilometres) that forms part of the Cantabrian Mountain Range, where there are many rivers with steep-sided valleys. Coastal humidity is held back by these mountains. Together these factors confer upon the area its particular microclimate, the details of which are provided in Supplementary Table S1 (data collected by an iMetos 2 agroclimate station present in Carballo since 2010).

### Botanical characterisation and data of agronomic interest

Plant characteristics were recorded over the growth cycle, collecting data on the features of the shoots, adult leaves, flowers and fruits. The flowers were inspected on 31st May 2018 (10 flowers collected from each of the three bushes). A botanical description was made following the protocol of the *International Union for the Protection of New Varieties of Plants*^15^ for the genus *Rosa*, as well as the descriptive method of Monserrat et al.^10^. Drawings and photographs of different plant organs were made. On the same 31st May, the number of flower buds, and the number of open roses per plant was recorded.

Between five and eight further roses were collected from each plant, and the number of petals per flower, the weight of the petals per flower and the weight of each petal recorded before freezing them at −30 °C until further analysis.

### Molecular characterisation

#### DNA extraction

Fresh leaves were collected in April 2018 and stored at −80 °C for DNA extraction. Using a pestle and mortar, leaf material (0.2 g) was homogenised in 1 mL of CTAB buffer, as described by De la Rosa et al.^28^. DNA was then extracted using the Maxwell^®^ PureFood Kit and Maxwell RSC Instrument (Promega Corporation, Madison, WI, USA) following the manufacture’s recommendations. The extracted DNA was resuspended in 100 µL of elution buffer (provided with the kit), and quantified using a Biodrop µLITE^®^ spectrophotometer (BioDrop, Cambridge, UK). DNAs extracted from the roses ‘Belle de Crécy’, ‘Jolande d’Aragone’, and ‘Alain Blanchard’ (Scariot et al.^17^) were used as reference for further analyses.

#### STMS amplification

STMSs were amplified by PCR in a 20 µL reaction volume containing 2 µL 10X PCR buffer (100 mM Tris-HCL, pH 8.3, 500 mM KCl), 1.5 mM MgCl_2_, 0.5 µM of each primer, 200 µM dNTP, 0.5 U taq-DNA polymerase (i.e., AmpliTaq Gold DNA polymerase [Applied Biosystems, Foster City, CA, USA]) and 50 ng of template DNA. Amplifications were performed in a PTC 100 thermocycler (MJ Research, Watertown MA, USA). The primers used, developed by Esselink et al.^29^, were RhAB22, RhE2b, RhD221, RhO517, and RhP519. The forward primers were labelled with a fluorochome (6-FAM, HEX or NED). Amplification cycles consisted of an initial step of 11 min at 95 °C, followed by 26 cycles of 30 s at 95 °C, 40 s at 55 °C, 1 min 30 s at 72 °C, with a final extension step of 45 min at 72 °C.

#### Detection of STMS polymorphism

One microlitre of a mix containing amplification products of three differently labelled loci was added to 3 µL of a mix containing 10:2:1 parts formamide, GeneScan-350ROX size standard (Applied Biosystems) and a loading buffer (25 mM EDTA, 50 mg mL^−1^ blue dextran). Fluorescent samples were denatured at 95 °C for 5 min and the DNA fragments separated on a sequencing gel (4.25% acrylamide, 1X TBE buffer, 6 M urea) using an ABI-PRISM 377 DNA sequencer running GeneScan software (Applied Biosystems).

#### Data analysis

The obtained STMS peaks were scored as discrete variables (namely allelic phenotype), using 1 or 0 to indicate the presence or the absence of each fragment. Data were compared with the original dataset reported by Scariot et al.^17^, made available by the authors. The genetic distance between pairs of accessions was estimated on the basis of the Nei coefficient and a principal coordinate analysis (PCA) was conducted using GeneAlEx 6.3^30^.

### Histological characterisation of the petals

Several petals from each of the three rosebushes were thawed to room temperature and fixed in FAA (90% 70° ethanol, 5% glacial acetic acid, 5% formaldehyde) for 48 h. They were then transferred to 70° alcohol, passed through a dehydrating series of ethanol solutions (using isoamyl acetate as an intermediary liquid) and set in paraplast blocks. These were cut using a 12 µm microtome and deposited on microscope slides. Some preparations were stained with safranin and fast green (before being immersed in xylene to remove the paraffin), mounted in Entellan, and examined using a Nikon E600 microscope (bright field, polarized light and epifluorescence microscopy).

For histochemical detection of lipids, small fragments of petals were fixed for 24 h in 4% paraformaldehyde in 0.1 M phosphate buffer (PB). The samples were rinsed with the same buffer and cryoprotected by passing through sucrose solutions in increasing concentration (10, 20, and 30%). Sections (12 µm thick) were obtained in a cryostat and collected on gelatin-coated slides. The stain was performed with the Sudan III technique following the standard method described in Kiernan^31^. The sections were examined with an Olympus BX51 microscope and photographed with an Olympus DP71 digital camera.

Fragments of the fixed petals were also passed through a dehydrating series of ethanol solutions and after critical point drying were gold-covered and examined using a FEI Quanta 600 environmental scanning electron microscope (ESEM).

### Analysis of petal volatile compounds

Six to eight more flowers from each of the three rosebushes were collected, their petals separated, weighed and stored separately (by bush) at −80 °C. A portion of those from each bush—Bush 1 = 30.46 g; Bush 2 = 38.33 g; Bush 3 = 39.90 g—was lyophilised using a Gamma 2–16 LSCplus lyophilising device (CHRIST, Osterode am Harz, Germany) at 0.10 mbars (lyophilised Bush 1 = 3.4 g; lyophilised Bush 2 = 4.67 g; lyophilised Bush 3 = 3.29 g) and sent for volatile compound analysis at the *Instituto de Ciencia y Tecnología de los Alimentos y Nutrición*-*CSIC* (Madrid, Spain).

The volatile compound profile of the essential oil of a Damask rose of French origin (Rose absolute, Moroccan, Ref.W298816-Sample-K) was examined as a reference against which to compare the studied petals. Samples for analysis were prepared in duplicate on the same day and maintained refrigerated until use. Samples of 0.05 g of each petal sample, or 20 µl of the Damask oil, were placed in a 20 mL glass headspace vial with a silicon/PTFE septum screw cap. Analyses were undertaken over two days, using one of the replicates each day, employing divinylbenzene/carboxen fibres (Ref. 57328-U Supelco [Merck KGaA, Darmstadt, Germany]). The volatile compounds of each were extracted by solid phase microextraction (SPME) and analysed by GC-MS (CG: Agilent 6890N device [Agilent Technologies, Santa Clara, CA, USA]; MS: 5973 device [Agilent Technologies, Santa Clara, CA, USA]) running MSD Chemstation software.

The technical details for CG-MS included equilibration of the heating plate at 50 °C for 30 min and 50 °C for 15 min extraction with the fibe; desorption of the injector at 240 °C for 15 min; volatile compounds column DB-WAXetr (polyethylene glycol 60 m, 320 × 0.25 μm); carrying gas - helium, constant flow (1.3 mL/min); Injection - desorption SPME splitless, 240 °C; heating gradient −40 °C 4 min, 4 °C/min 110 °C, 6 °C/min 180 °C, 8 °C/min 240 °C 15 min; auxiliary temperature 250 °C, detector temperature 230 °C; mass range (*m/z*)—27–450; data treatment: MassHunter Qualitative Analysis B.07.00 software.

Compounds were identified by comparison against the spectra in the Wiley Registry 7th Edition Spectral Library and the National Institute of Standards and Technology 2008 Mass Spectral Library (NIST 08), and by calculating linear retention indices with respect to a series of alkanes (C6-C20).

## Results

### Botanical characterisation and data of agronomic interest

**Fig. 1:**
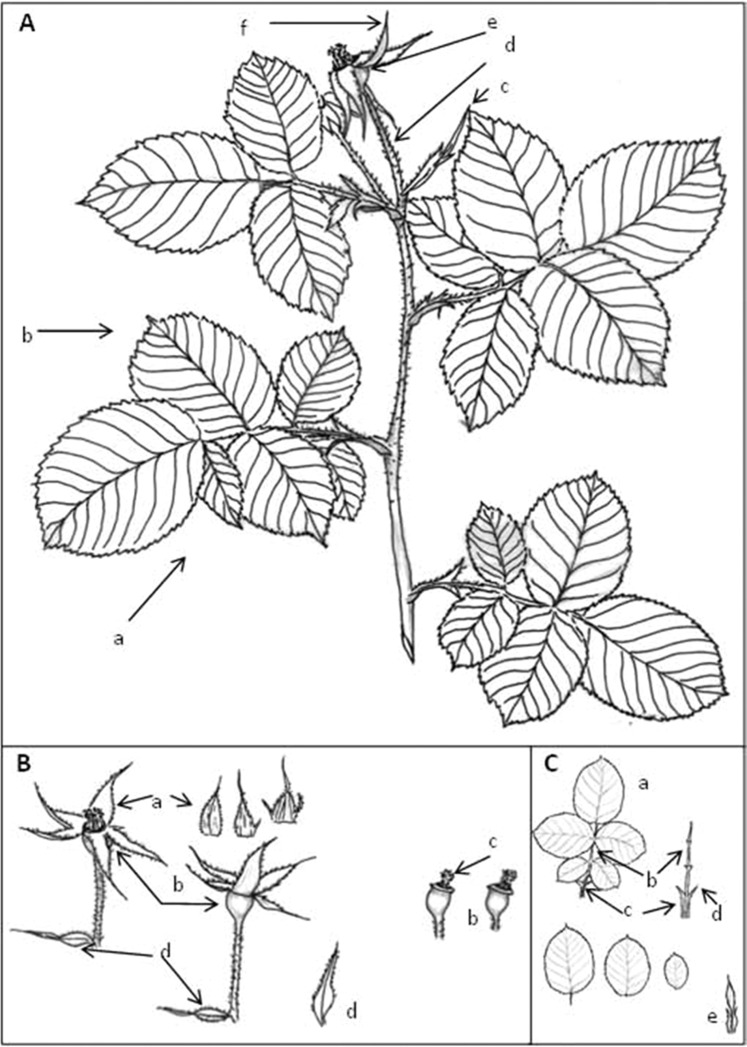
Details of different parts of the leaves and flowers of the Narcea variety. **a** Inflorescence with leaves (a) folioles (b) bract (c) pedicel (d) hypanthium (e), sepals (f). (**b**) (a) sepals (b) hypanthium (c) stamens (d) bract. (**c**) Folioles (a) united by a central vein or rachis (b) at the base of which are stipules (c) fused to the peciole except on the upper part or auricle (d). Left. bract (e) of the flower

**Fig. 2:**
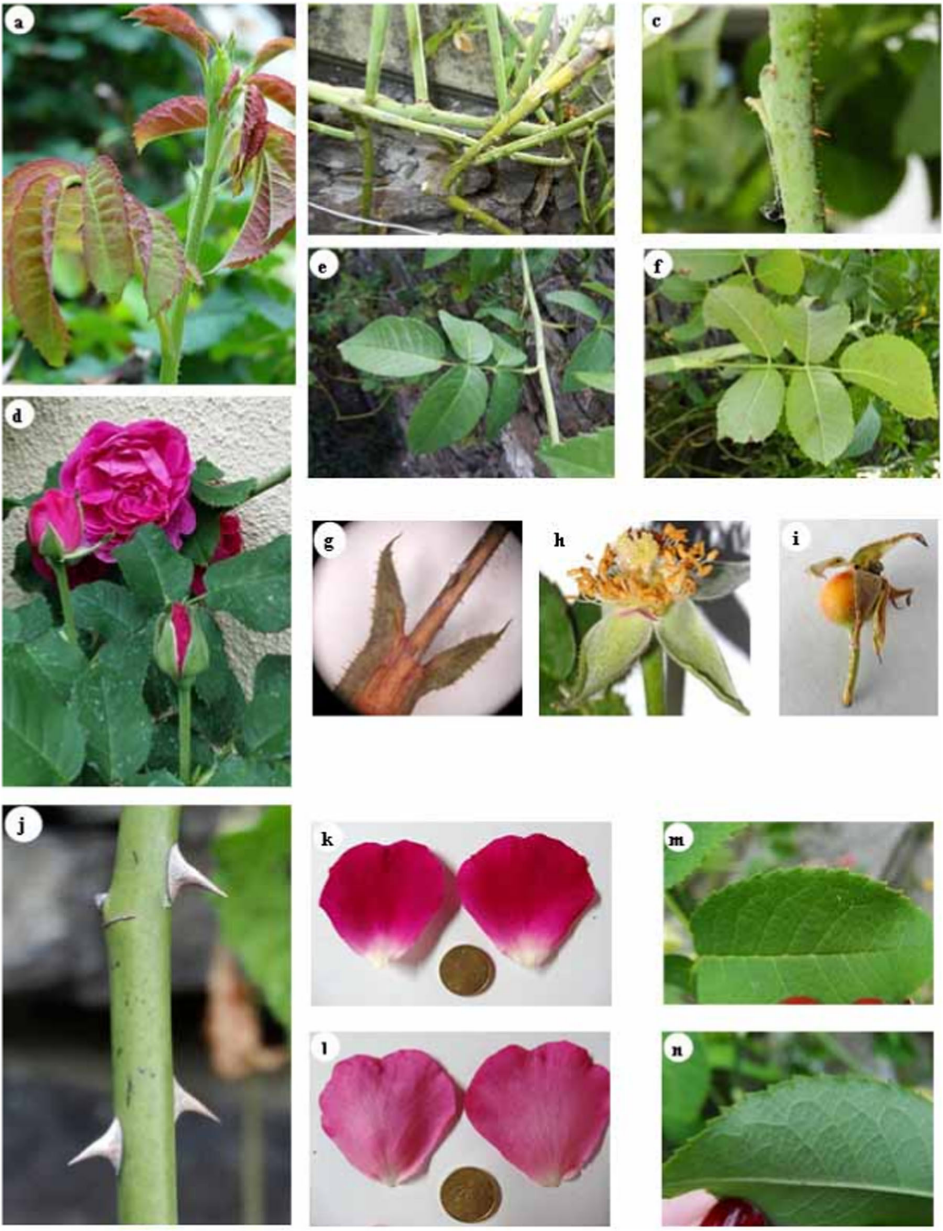
Details of different organs of the *Narcea* variety. Shoot with copper-coloured leaves (**a**), dispersed thorns (**b**), narrow, straight spines (**c**), “double” roses with purplish-red flowers (**d**), leaves with 5 folioles, upper surface (**e**), lower surface (**f**), stipule (**g**), stamens and pistil (**h**), false fruit or rose hip (**i**), upper side of petal (**k**), lower side of petal (**i**), upper side foliole (**m**), lower side foliole (**n**). Reference: 10 €cent coin

The following is a description of the examined rosebushes according to the descriptive method of Montserrat el al.^11^ (Figs. 1 and 2). Bush with strong, erect stems, 2–3 m long. *Thorns* are very dispersed, yellowish, triangular (isosceles) becoming quickly wider towards the base; point straight or slightly curved. Thorns interspersed by numerous straight, narrow spines. *Leaves* 10–13 × 8.5–11 cm, with 5 leaflets (sometimes three at the base of the inflorescence) of 5 × 3.5–4 cm (the terminal leaflet is larger with a longer point, sharp or slightly acuminate), thick, ovate, coriaceous, upper and lower surfaces glabrous, discoloured with a few sessile glands on the rachis; denticulation not deep, crenated, almost simple without glands. *Stipules* long and narrow (2.5 × 0.2 cm), concrescent with peciole and with two pointy lobules, divergent, nearly 1 cm in length, hairy and with pectin glands along the margin. *Inflorescence* with one, sometimes a few, large (8 cm diameter) fragrant flowers with very fallen bracts and a long pedicel (2–3 cm), thorn-covered and glandular. *Petals* purplish-red, styles free but generally grouped into a column that does not pass beyond the line of the stamens. *Stylar disc* a little concave, 6.70 mm in diameter with a pore 2.95 mm in diameter; the disc size is therefore large and the pore size small (33–50% of the disc size). *Sepals* triangular, prolonged, with an apical lobule, hardly any lateral projections, reflexing and persistent during fruiting, dorsal and lobules glandulous. *Fruit* subglobous or slightly urceolate, a little over 1.2 cm long and 1.1 cm wide at the broadest point.

The flowers are very attractive given their intense fragrance and the homogenous purplish-red colour of their many medium-large sized petals (those at the centre are smaller) (Supplementary Fig. S2).

Supplementary Table S2 shows the full botanical characterisation results according to the International Union for the Protection of New Varieties of Plants^15^ protocol. Supplementary Table S3 relates the botanic features of agronomic interest.

### Molecular characterisation

Microsatellite profiles of Narcea rose and reference genotypes (‘Belle de Crécy’, ‘Jolande d’Aragone’, and ‘Alain Blanchard’) were examined and compared with those of 44 ancient garden roses representative of the main horticultural groups (3 Alba, 3 Hybrid China, 3 Noisette, 4 Tea, 5 Bourbon, 4 Centifolia, 2 Damask, 8 Gallica, 2 Hybrid Perpetual, 3 Moss, and 7 Portland), previously obtained by Scariot et al. (2006)^17^.

**Fig. 3:**
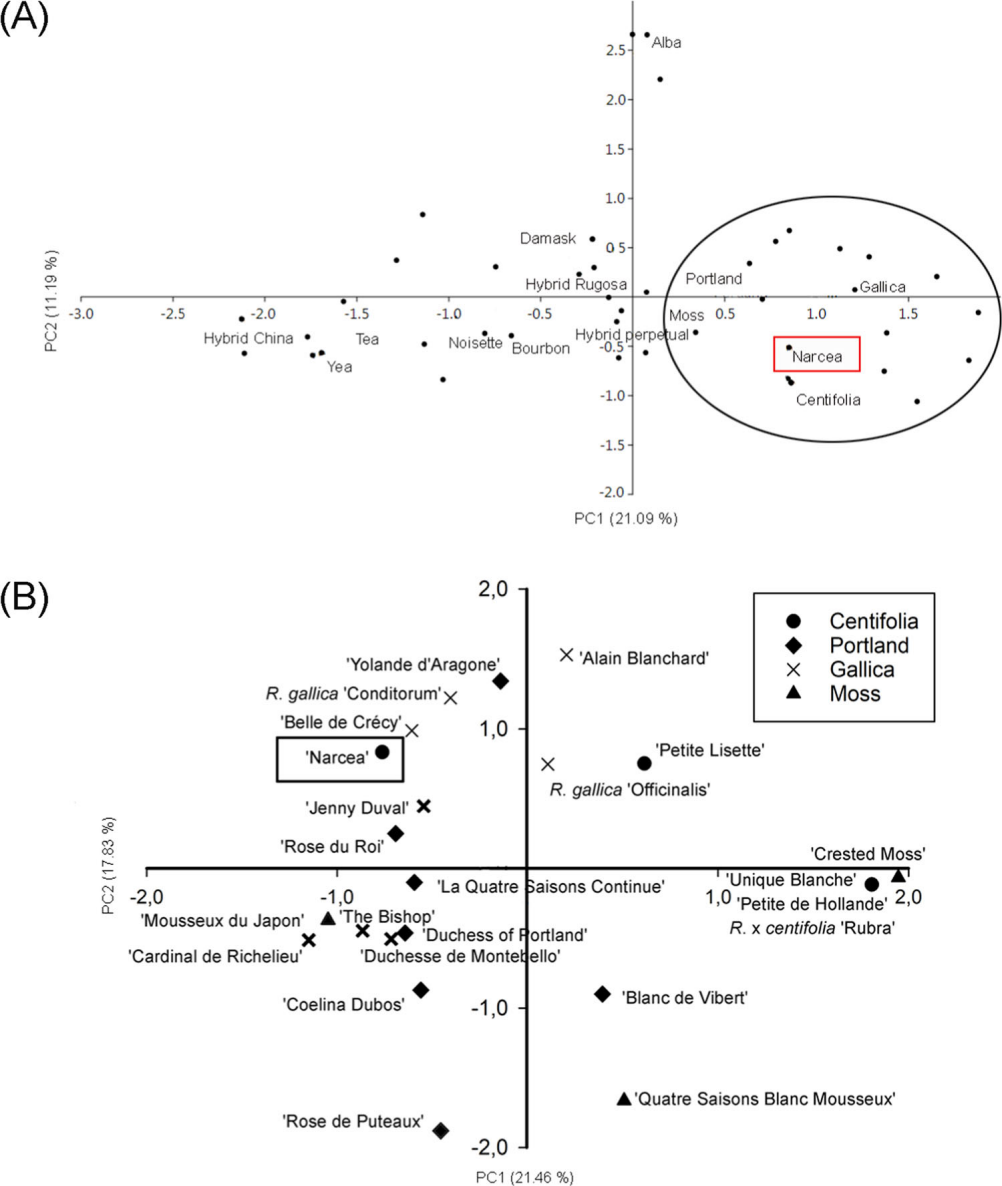
**a** Principal coordinates analysis scatter diagram for the *Narcea* and rose horticultural group (Scariot et al.)^17^: STMSs data. **b**. Principal coordinates analysis scatter diagram for the *Narcea* and rose cultivars belonging to the horticultural groups Gallica, Centifolia, Moss and Portland (Scariot et al.)^17^: STMSs data

The genetic relationships are illustrated via PCA scatter plots (Fig. 3). The first two coordinates in Fig. 3a account for 21.09 and 11.19% of the variance. With high first axis values, the examined material fell between the rose groups Centifolia, Gallica, Moss, and Portland. An in-depth analysis was therefore performed to better understand the relationships between the examined material and the latter groups. Figure 3b shows the examined material to cluster within the Gallica group, and to be particularly close to the cultivar ‘Belle de Crécy’ and ‘Jenny Duval’ whose allele sizes are reported in Table 1.

**Table 1:** Allele sizes (bp) of the STMS markers used to characterize the unknown ancient cultivated rose variety (Narcea), ‘Belle de Crécy’, and ‘Jenny Duval’ as reported by Scariot et al.^17^

Allele sizes (bp)			
STMS locus	‘Narcea’	‘Belle de Crécy’	‘Jenny Duval’
RhAB22	165, 169	165, 169	165
RhD221	206, 217, 220, 223	220	206, 220
RhE2b	179, 185, 189	173, 182, 185	176, 185
RhO517	251, 254, 257, 260	257, 260	254, 257, 260
RhP519	217, 229, 235	217, 229, 238	217, 229

### Histological characterisation of the petals

**Fig. 4:**
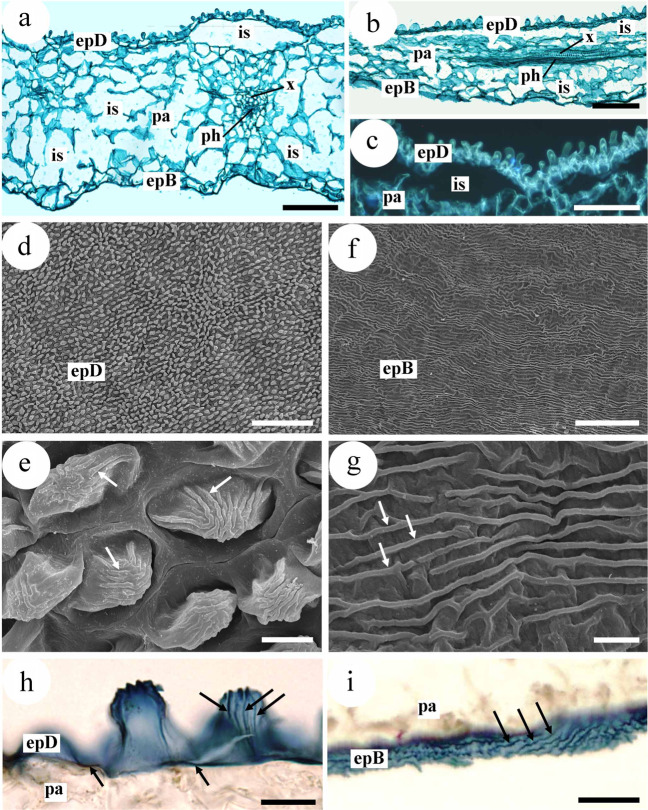
Histological study of a *Narcea* rose petal. **a** Transverse section of the petal. Between the adaxial (epD) and abaxial epidermises (epB) lies storage parenchyma (pa) with many intracellular spaces (is). Notice also xylem (x) and phloem (ph) making up a small, collateral vascular bundle. Note also the papillae on the adaxial epidermal cells. **b** Longitudinal section of the lamina. **c** Detail of the adaxial epidermis (epD) with papilliform cells of the adaxial surface. **d** Abundant promontories corresponding to the papillae of the epidermal cells. **e** Detail of ‘**d**’. The arrows mark striations in the cuticle of the epidermal cells. **f**–**g** Surface of abaxial epidermis. **f** Note the epidermal cells are not papilliform. **g** Detail of latter. Arrows mark cuticular striations. **h**, **i** Detection of lipids. **h** Lipids (arrows) in the adaxial epidermis (epD), in fact in the cuticular striations and the spaces between the papillae. **i** Lipids (arrows) in the striations of the abaxial epidermis (epB). **a**, **b** safranin and fast green. **h**, **i** Sudan III. **a**, **b** **h**, **i** Optical microscopy, bright field. **c** Epifluorescence microscopy. **d**–**g** Scanning electron microscopy. *epB* epidermis abaxial, *epD* epidermis adaxial, *is* intercellular space, *pa* storage parenchyma, *ph* phloem, *x* xylem. Bars: **a**–c = 100 µm, **d** = 200 µm, **e** = 10 µm, **f** = 50 µm, **g** = 5 µm, **h**, **i** = 20 µm

Figure 4 shows (from the adaxial to the abaxial face of the leaf) the different parts of the studied petals mesophyll. The adaxial epidermis is formed by a single layer of papilliform cells (Fig. 4a–c). These papillae afford the epidermis a granular appearance (Fig. 4d). Cuticular striations converge towards the apex of these papillae (Fig. 4e). Lipids were visible in the spaces between the papillae and on these striations (Fig. 4h). The storage parenchyma shows abundant, large intercellular spaces (Fig. 4a–c) with no crystalline inclusions. Small collateral vascular bundles are also visible (Fig. 4a, b).

The abaxial epidermis is a single layer of flat cells (Fig. 4a, b). The cuticle has striations that do not mark the edges of cells (Fig. 4f, g); these striations contain lipids (Fig. 4i).

### Petal volatile compounds

**Fig. 5:**
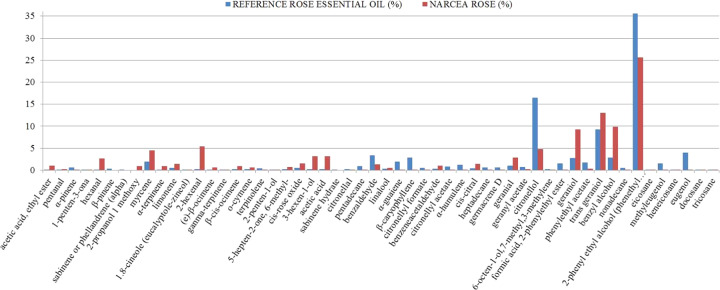
Percentage of different volatile compounds in *Narcea* petals compared to the reference rose essential oil (Rose absolute. Moroccan. Ref.W298816-Sample-K)

Supplementary Table S4 shows the volatile compounds identified, ordered by family and indicating the retention times (RT), the calculated linear retention indices (LRIc), and the same index as recorded in the available libraries NIST 08 and Wiley (LRIl). Supplementary Table S5 and Fig. 5 show the quantities of different compounds detected in the examined petals (expressed as a percentage of the total compounds detected) compared to the profile of the commercial rose essential oil. These results do not preclude the presence of other, minor compounds. The values in Supplementary Table S5 are the means of the two measurements made for each petal analysed, and in the case of the oil, of the duplicate analysis.

## Discussion

The present results show the examined material to belong to an unknown, ancient cultivated variety of rose, for which the name *Narcea* is here proposed in honour of the geographical area of the Principality of Asturias (northern Spain) where the rose was discovered.

Both the botanical description undertaken following the method of Montserrat et al.^11^, and of the International Union for the Protection of New Varieties of Plants^16^, showed the studied material to have characteristics common to both *R. gallica* and *R. centifolia* (as described by Decaisne and Naudin^3^), including its intense aroma, the presence of glandulous hairs, and leaves with five folioles. It also had some characteristics exclusive to *R. gallica*, such as inflorescences formed by a single (or sometimes a few) erect, purplish-red aromatic flowers, and styles with yellowish hairs grouped into a column. Note that *R. centifolia* has hanging flowers, with clear pink petals, and the calyx tube has reddish hairs.

The studied material was also similar to *R. gallica* in terms of its little-concave disc, the presence of triangular, reflexing sepals with hardly any lateral projections, and with a globous dorsal side and lobules, as well as the subglobous form of the fruit.

Supplementary Table S3 shows the examined material’s features of agronomic interest. The mean number of open roses for the three bushes was 37.33, although the coefficient of variation was large (as determined on 31st May). The number of closed buds on each bush was even more variable (between 11 and 130). This variation might be explained by the fact that one of the bushes was planted in the ground and the other two in large pots, which could have influenced their vigour and therefore the number of buds and open flowers produced. For more reliable data regarding these variables, studies on larger numbers of bushes of the same age would be required, with all subjected to the same cultivation practices. Both, the total number of petals and the flower weight are higher in Rosa Narcea than in *R. damascena* and *R. centifolia*^32,33^. Also, the mean petal weight of 10.43 g/rose was rather constant. Thus, being in the ground or in a pot seems to influence the number of flowers produced, but not the weight of the petals of those flowers—a character apparently more strongly related to varietal identity than the conditions under which plants grow. The mean number of petals per rose was also similar across the bushes (around 62 petals per rose). The mean petal weight per flower was 0.26 g, but in this case the coefficient of variation was larger. Indeed, Fig. 4 shows that the petals were not all of the same size, with the three of four at the centre surrounding the reproductive organs much smaller than the rest.

The results of the STMS analysis (Table 1 and Fig. 3) show that Narcea most likely is a tetraploid genotype as well as *R*. x *centifolia* and *R*. *gallica*^34^ and is genetically close to the Gallica cultivars ‘Belle de Crécy' and ‘Jenny Duval’, with unknown parents. Many modern roses are also tetraploids^9,21^ and all the essential oil Rosa species/cultivars are tetraploid (2n = 4× = 28), providing better chance for successful crosses^35^.

The examined petals had the same general histological features of all petals of the genus *Rosa*^36–39^, e.g., an adaxial epidermis with papilliform cells, an abaxial epidermis in which these cells are absent, with both epidermises showing striations, and the storage parenchyma with abundant spaces.

The histochemical location of the lipids in the epidermis, and their absence in the intercellular spaces may reflect the functional moment of the petal. Petals mostly emit their fragrance when the flower is fully open^37^; this is when the intercellular space contains droplets of essential oil^39^. However, if oil does not enter the intercellular spaces until a certain time of day, say the afternoon or evening, this would explain its absence in the examined petals (the flowers were picked in the morning). The molecules that provide the characteristic smell are released to the exterior via the cuticle^37,40^; certainly, oils were detected in the cuticles of both epidermises.

Figure 5 and Supplementary Table S5 show the concentrations of volatile compounds detected in the petals. Compounds such as nonadecane, methyleugenol, heneicosane and tricosane, which reduce the quality of rose essential oil^27,41^ were absent or in very small quantities in the examined material. The examined material contained a number of compounds of great interest in perfume and rose essential oil production, such as hexanal, myrcene, α-terpinene, limonene, 2-hexenal, (e)-β-ocimene, β-cis-ocimene, o-ocymene, 5-hepten-2-one, 6-methyl-, cis-rose oxide, 3-hexen-1-ol, cis-citral (-neral or 2,6-octadienal, 3,7-dimethyl), and especially geranial (alpha-citral or trans), geraniol, trans geraniol and benzyl alcohol.

The key aroma compounds of rose based products are β-phenylethyl alcohol, citronellol, geraniol, eugenol, linalool and rose oxide^40^, and all are present in the petals from Narcea Rose. The compounds cis-rose oxide, cis-citral, geranial, geraniol and trans geraniol, which are found in high quality oil^27,41^, were present in large amounts in the examined petals. However, citronellol appeared in lower proportion than in the reference essential oil. Cis-rose oxide, cis-citral, geranial, geraniol, tras geraniol and citronellol together confer cut grass-type aromas to different types of fruit (e.g., green apple), jasmine flowers and rose flowers. Their varying presence and proportion in different rose essential oils determines their character and quality. Other components derivatives of monoterpene alcohols as Citronellyl formate, citronellyl acetate, geranyl acetate and neryl acetate are, which made the aroma more harmonious^24^ are also present in the examined petals.

It should be remembered that a rose’s aroma is not the product of a single compound but a complex mixture of compounds, some of which may be present in tiny amounts^42^. It is precisely this complexity that makes it so hard to synthesise an alternative in the laboratory. Rose essential oil - the most expensive of all plant essential oils—has, therefore, to be made from rose petals. Obtaining one litre of *R. damascena* or *R. centifolia* oil may require 3–4 tons of petals^26^, all of which need to be picked over a single, relatively short flowering period (end May-early June), explaining why that litre may fetch €14,000. Fortunately, only tiny quantities are needed to produce much larger volumes of perfume and other cosmetics. The discovery of the *Narcea* variety may, therefore, have an important economic dimension as there is need to develop the new varieties of oil-bearing roses by maintaining the standards of scent molecules of rose oil. But among all Rosa species, about 20% species are considered as scented, 50% are low scented and the rest are non-scented^35^ and there are a lot of steps required creating a new cultivar from traditional crosses^35^. Flower morphology and scent composition of the Narcea Rose show that this particular rose has an important breeding potential.

The variety may also be of pharmacological, medical and food interest. Certainly, ancient cultures used roses for the treatment of many physical and mental problems^43–47^, and in recent years interest has developed in understanding the true medical and food-value properties of these plants^48,49^. Further work is needed to determine whether this is the case.

## Conclusions

The present results show the discovered rosebushes to belong to an unknown, ancient cultivated variety, for which the name *Narcea* is proposed. A complete description is provided of the first reliably recorded ancient, cultivated rose from Spain. The present work is the first step required for the variety’s protection and official recognition as a new, Spanish genetic resource. Some of the results suggest the variety may be of interest to the perfume industry. More work is needed to determine the best way to cultivate this variety and to further explore its agricultural and industrial potential.

## Supplementary information

SUPPLEMENTARY TABLES AND FIGURES

## Data Availability

The data supporting the results presented in this work are available in the supplementary information
