# Supplementary material for: Narcea—an unknown, ancient cultivated rose variety from northern Spain
Source: Hortic Res. 2020 Apr 1;7:44. doi: 10.1038/s41438-020-0266-8 (PMC7109042; doi:10.1038/s41438-020-0266-8)
Supplement: Supplementary file 1 — SUPPLEMENTARY TABLES AND FIGURES [file 41438_2020_266_MOESM1_ESM.docx]

**SUPPLEMENTARY MATERIAL/INFORMATION**

**SUPPLEMENTARY TABLES**

**Supplementary Table S1.** Climate data (mean values) for the area where the unknown, ancient cultivated rose variety (*Narcea*) was discovered.

| **Variable** | **Years**  **(2010-2017)** | **May**  **(2010-2017)** | **June**  **(2010-2017)** | **Year 2018** | **May**  **2018** | **June 2018** |
| --- | --- | --- | --- | --- | --- | --- |
| **Air temp (ºC)** | 12.38 | 13.05 | 16.51 | 12.20 | 12.62 | 16.22 |
| **Max. air temp (ºC)** | 19.72 | 19.99 | 23.86 | 18.79 | 19.20 | 22.16 |
| **Min. air temp (ºC)** | 4.16 | 7.89 | 11.05 | 7.47 | 7.83 | 12.24 |
| **Rainfall (mm)** | 1199.54 | 83.73 | 77.09 | 1488.00 | 106.60 | 168.60 |
| **Rel. humidity (%)** | 77.95 | 75.16 | 77.15 | 79.81 | 82.37 | 86.76 |
| **Leaf wetting (min)** | 505.04 | 537.78 | 473.87 | 399.42 | 375.48 | 329.83 |

**Supplementary Table S2**. UPOV (*Rosa* L.) characteristics for the unknown, ancient cultivated rose variety (*Narcea*).

| **UPOV Number^a^** | **Variable** | **Expression level** | **Note** |
| --- | --- | --- | --- |
| 1, (*), PQ, G, P | Plant: Growth type | shrub | 4 |
| 2, (*), (+), QN, G, P | Excluding varieties with growth type climber: Plant: growth habit | upright | 1 |
| 3, QN, C, G | Plant: height (during second flush) | tall | 7 |
| 4, (+), QL | Young shoot: anthocyanin coloration | present | 9 |
| 5, (+), QN | Young shoot: intensity of anthocyanin coloration | medium | 5 |
| 6, QN | Stem: number of prickles (excluding very small and hair like prickles) | medium | 5 |
| 7, PQ, (a) | Prickles: predominant colour (as for 6) | yellowish | 2 |
| 8, QN, (a) | Leaf: size | 12 cm length x 9.5 cm width | 5 |
| 9, QN, (a) | Leaf: intensity of green colour (upper side) | medium | 5 |
| 10, QL, G, P, (a) | Leaf: anthocyanin coloration | absent | 1 |
| 11, (*), QN, (a) | Leaf: glossiness of upper side | medium | 5 |
| 12, (*), QN, (a) | Leaflet: undulation of margin | absent or very weak | 1 |
| 13, (*), PQ, (a) | Terminal leaflet: shape of blade | medium elliptic | 2 |
| 14, (+), PQ, C, (a) | Terminal leaflet: shape of base of blade | cordate | 4 |
| 15, (+), PQ, (a) | Terminal leaflet: shape of apex of blade | acute | 2 |
| 16, (+), QL, G, P | Flowering shoot: flowering laterals | absent | 1 |
| 17, (+), QN, G, P | Flowering shoot: number of flowering laterals | very few | 1 |
| 18, (+), G, P | Only varieties with no flowering laterals: flowering shoot: number of flowers | few | 3 |
| 19, (+), QN, G, P | Only varieties with flowering laterals: flowering shoot: number of flowers per lateral |  |  |
| 20, (+), PQ, G, P | Flower bud: shape in longitudinal section | medium ovate | 2 |
| 21, (*), (+), QN, (b) | Flower: type | double | 3 |
| 22, (*), Qn, (b) | Flower: number of petals | many | 7 |
| 23, (*), (+), PQ, (b) | Flower: colour group | red purple D 181 O-H Magenta | 12 |
| 24, (+), PQ, G, (b) | *Only varieties with flower type: double flower*: colour of centre | red-purple D 181 O-H Magenta | 5, 6 |
| 25, QN, G, P, (b) | *Only varieties with flower type: double*: flower: density of petals | medium | 5 |
| 26, (*), QN, (b) | Flower: diameter | large, 7,90 cm | 7 |
| 27, (*), (+), PQ, (b) | Flower: shape | round | 1 |
| 28, (+), PQ, C, G, (b) | Flower: profile of upper part | flat | 1 |
| 29, (*), (+), PQ, C, G, (b) | Flower: profile of lower part | flattened convex | 3 |
| 30, QN, (b) | Flower: fragrance | strong | 3 |
| 31, (*), (+), QN, (b) | Sepal: extensions | weak | 3 |
| 32, (+), QL, (b),(c) | Petals: reflexing of petals one-by-one | absent | 1 |
| 33, (*), PQ, (b), (c) | Petal: Shape | obcordate | 4 |
| 34, QN, (b), (c) | Petal: incisions | weak | 3 |
| 35, QN, (b), (c) | Petal: reflexing of margin | absent or very weak | 1 |
| 36, QN, (b), (c) | Petal: undulation | absent or very weak | 1 |
| 37, (*), QN, G, P, (b), (c) | Petal: size | medium-large | 5, 7 |
| 38, (*), QN, C, (b), (c) | Petal: length | medium-long 4,90cm | 5, 7 |
| 39, (*), Qn, C, (b), (c) | Petal: width | medium-board 5,06cm | 5, 7 |
| 40, (*), QL, (b), (c) | Petal: number of colours on inner side (basal spot excluded) | one | 1 |
| 41, (*), QN, (b), (c) | *Only varieties with one colour on inner side of petal* - Petal: intensity of colour (basal spot excluded) | even | 2 |
| 42, (*), PQ, (b), (c) | Petal: main colour on the inner side (main colour is that with largest surface area), RHS Colour Chart (Indicate reference number) | RHS: D 181 O-H Magenta |  |
| 43, (*), PQ,(b), (c) | *Only varieties with two or more colours on inner side of petal*: Petal secondary colour (basal spot excluded) RHS Colour chart7 |  |  |
| 44, PQ, (b), (c) | *Only varieties with more than two colours on inner side of petal*: Petal: tertiary colour (basal spot excluded) |  |  |
| 45, (*), (+), PQ, (b), (c) | *Only varieties with two or more colours on inner side of petal*: Petal: distribution of secondary colour on inner side (basal spot excluded) |  |  |
| 46, (+), PQ, (b), (c) | *Only varieties with more than two colours on inner side of petal*: Petal: distribution of tertiary colour on inner side (basal spot excluded) |  |  |
| 47, (*), QL, (b), (c) | Petal: basal spot on the inner side | present | 9 |
| 48, (*), (+), QN, (b), (c) | Petal: size of basal spot on inner side | small | 3 |
| 49, (*), PQ, (b), (c) | Petal: colour of basal spot on inner side | white | 1 |
| 50, (*), PQ, (b), (c) | Petal: main colour on the outer side (only if clearly different from inner side) | RHS: E29 Schev. Rose Deep |  |
| 51, PQ, (b) | Outer stamen: predominant colour of filament | white | 1 |
| 52, QN, G | Seed vessel: size (at petal fall) | medium | 5 |
| 53, (+), PQ, G | Hip: shape in longitudinal section | pitcher-shaped | 2 |
| 54, (+), PQ, G | Hip: colour (at mature stage) | yellow | 1 |

^a^ (*) Features included in the examination instructions for the UPOV international harmonisation of the description of varieties. QL: qualitative character; QN: quantitative character; PQ: pseudoqualitative characters; (a) - (c): observations relative to several characters; (+): explanations relative to single characters; C: cur flow; G: Ground-planted rosebush; P: Potted rosebush

**Supplementary Table S3.**Values of variables of agronomic interest for the unknown, ancient cultivated rose variety (*Narcea*).

|  | Mean | Standard deviation | Coefficient of variation (%) |
| --- | --- | --- | --- |
| Nº open roses (31^st^ May) per plant | 37.33 | 9.81 | 26.27 |
| Nº unopened buds (31^st^ May) per plant | 53.67 | 66.26 | 123.45 |
| Nº petals/rose | 62.16 | 8.06 | 12.96 |
| Weight of petals per rose (g) | 10.43 | 2.01 | 19.27 |
| Petal weight (g) | 0.26 | 0.09 | 35.07 |

**Supplementary Table S4.**Volatile compounds detected (by family). RT: retention time (mins); LRIc: calculated linear retention rime; LRIl: linear retention time as recorded in the available libraries NIST 08 and Wiley (LRIl).

| **Compound identified** | **RT** | **LRIc** | **LRIl** |
| --- | --- | --- | --- |
| **Acids** |  |  |  |
| acetic acid | 24.474 | 1504 | 900 |
| **Alkanes** |  |  |  |
| pentadecane | 25.767 | 1543 | 1500 |
| heptadecane | 30.763 | 1723 | 1700 |
| nonadecane | 34.74 | 1923 | 1900 |
| eicosane | 36.092 | 2001 | 2000 |
| heneicosane | 37.59 | 2201 | 2100 |
| docosane | 38.951 | 2301 | 2200 |
| tricosane | 40.224 | 2395 | 2300 |
| **Alcohols** |  |  |  |
| 2-propanol 1 methoxy | 13.096 | 1130 |  |
| 2-penten-1-ol | 20.157 | 1400 | 1326 |
| 3-hexen-1-ol | 22.386 | 1459 | 1418 |
| Benzyl alcohol | 34.213 | 1893 | 1902 |
| 2-phenyl ethyl alcohol (phenethyl alcohol) | 35.096 | 1943 | 1912 |
| methyleugenol | 36.433 | 2024 | 2033 |
| eugenol | 38.761 | 2287 | 2172 |
| **Aldehydes** |  |  |  |
| pentanal | 8.338 | 920 |  |
| hexanal | 11.661 | 1016 | 1020 |
| 2-hexenal | 16.609 | 1242 | 1220 |
| benzaldehyde | 26.645 | 1570 | 1520 |
| benzeneacetaldehyde | 29.573 | 1674 | 1656 |
| **Ketones** |  |  |  |
| 1-penten-3-ona | 9.582 | 931 | 1013 |
| 5-hepten-2-one, 6-methyl- | 20.839 | 1427 | 1350 |
| **Esters** |  |  |  |
| acetic acid, ethyl ester | 6.123 | 917 | 900 |
| formic acid, 2-phenylethyl ester | 32.588 | 1809 | 1768 |
| phenylethyl acetate | 33.135 | 1837 | 1822 |
| **Monoterpene hydrocarbons** |  |  |  |
| α-pinene | 9.490 | 929 | 936 |
| β-pinene | 12.378 | 1111 | 1097 |
| sabinene | 12.925 | 1125 | 1115 |
| β-phellandrene (alpha) | 12.925 | 1125 | 1203-1160 |
| myrcene | 14.579 | 1169 | 1163 |
| α-terpinene | 15.110 | 1183 | 1175 |
| **Monoterpene hydrocarbons** |  |  |  |
| limonene | 15.789 | 1201 | 1194 |
| 1.8-cineole (eucalyptole-zineol) | 16.052 | 1215 | 1206 |
| (e)-β-ocimene | 17.214 | 1273 | 1251 |
| γ-terpinene | 17.506 | 1287 | 1243 |
| β-cis-ocimene | 17.809 | 1320 | 1244 |
| o-cymene | 18.546 | 1345 | 1268 |
| terpinolene | 18.907 | 1366 | 1281 |
| **Oxygenated terpenes** |  |  |  |
| citronellal | 25.343 | 1530 | 1464 |
| linalool | 27.026 | 1582 | 1553 |
| citronellyl formate | 28.909 | 1649 | 1628 |
| citronellyl acetate | 29.827 | 1684 | 1658 |
| cis-citral | 30.320 | 1703 | 1663 |
| geranial | 31.393 | 1752 | 1719 |
| geranyl acetate | 31.876 | 1775 | 1759 |
| citronellol | 32.130 | 1787 | 1774 |
| geraniol | 32.744 | 1817 | 1818 |
| trans geraniol | 33.701 | 1866 | 1861 |
| **Hydrocarbonated sesquiterpenes** |  |  |  |
| α-guaiene | 28.304 | 1626 | 1621 |
| β-caryophyllene | 28.465 | 1632 | 1601 |
| α-humulene | 30.158 | 1696 | 1660 |
| germacrene D | 31.022 | 1735 | 1690 |
| **Other monoterpenes** |  |  |  |
| cis-rose oxide | 21.279 | 1437 | 1363 |
| sabinene hydrate | 24.801 | 1514 | 1456 |

**Supplementary Table S5.**Mean percentage value of volatile compounds in the petals of the unknown, ancient cultivated rose variety *Narcea*, and of the essential oil 'Rose absolute, Moroccan, Ref.W298816-Sample-K'.

| **COMPOUND** | **Essential oil profile (%)^a^** | | | ***Narcea rose*(%)** | | |
| --- | --- | --- | --- | --- | --- | --- |
|  | Mean | SD | CV % | Mean | SD | CV% |
| acetic acid, ethyl ester | 0.028 | 0.001 | 3.82 | 1.012 | 0.202 | 19.95 |
| pentanal | 0.020 | 0.001 | 6.41 | 0.287 | 0.097 | 33.78 |
| α-pinene | 0.676 | 0.042 | 6.17 | 0.002 | 0.000 | 19.75 |
| 1-penten-3-ona | 0.019 | 0.000 | 2.58 | 0.049 | 0.007 | 14.00 |
| hexanal | 0.023 | 0.001 | 5.62 | 2.716 | 0.495 | 18.23 |
| β-pinene | 0.375 | 0.025 | 6.56 | 0.000 | 0.000 | 0.00 |
| sabinene o phellandrene (alpha) | 0.155 | 0.005 | 3.19 | 0.000 | 0.000 | 0.00 |
| 2-propanol 1 methoxy | 0.000 | 0.000 | 0.000 | 0.927 | 0.283 | 30.51 |
| myrcene | 1.974 | 0.085 | 4.30 | 4.467 | 0.407 | 9.11 |
| α-terpinene | 0.061 | 0.001 | 1.75 | 0.906 | 1.170 | 129.05 |
| limonene | 0.576 | 0.003 | 0.54 | 1.447 | 0.068 | 4.66 |
| 1.8-cineole (eucalyptole-zineol) | 0.034 | 0.001 | 3.31 | 0.068 | 0.012 | 17.26 |
| 2-hexenal | 0.237 | 0.009 | 3.93 | 5.461 | 0.654 | 11.99 |
| (e)-β-ocimene | 0.120 | 0.006 | 5.00 | 0.679 | 0.066 | 9.73 |
| γ-terpinene | 0.114 | 0.008 | 7.22 | 0.042 | 0.003 | 7.66 |
| β-cis-ocimene | 0.254 | 0.004 | 1.63 | 0.987 | 0.107 | 10.82 |
| o-cymene | 0.259 | 0.007 | 2.89 | 0.612 | 0.142 | 23.13 |
| terpinolene | 0.398 | 0.021 | 5.35 | 0.185 | 0.019 | 10.06 |
| 2-penten-1-ol | 0.040 | 0.001 | 2.49 | 0.185 | 0.025 | 13.45 |
| 5-hepten-2-one, 6-methyl- | 0.235 | 0.012 | 5.12 | 0.779 | 0.165 | 21.22 |
| cis-rose oxide | 0.525 | 0.025 | 4.77 | 1.567 | 0.122 | 7.78 |
| 3-hexen-1-ol | 0.214 | 0.005 | 2.49 | 3.139 | 0.629 | 20.05 |
| acetic acid | 0.222 | 0.010 | 4.66 | 3.169 | 0.436 | 13.77 |
| sabinene hydrate | 0.098 | 0.002 | 2.34 | 0.000 | 0.000 | 0.00 |
| citronellal | 0.263 | 0.011 | 4.13 | 0.078 | 0.005 | 6.45 |
| pentadecane | 1.003 | 0.012 | 1.23 | 0.067 | 0.013 | 19.62 |
| benzaldehyde | 3.401 | 0.057 | 1.69 | 1.402 | 0.144 | 10.29 |
| linalool | 0.350 | 0.008 | 2.19 | 0.514 | 0.090 | 17.54 |
| α-guaiene | 2.002 | 0.031 | 1.57 | 0.061 | 0.021 | 34.79 |
| β-caryophyllene | 2.922 | 0.061 | 2.08 | 0.014 | 0.005 | 32.07 |
| citronellyl formate | 0.577 | 0.017 | 2.91 | 0.022 | 0.006 | 29.49 |
| benzeneacetaldehyde | 0.299 | 0.001 | 0.31 | 1.029 | 0.088 | 8.59 |
| citronellyl acetate | 0.842 | 0.005 | 0.61 | 0.057 | 0.009 | 16.22 |
| α-humulene | 1.225 | 0.016 | 1.27 | 0.012 | 0.002 | 15.77 |
| cis-citral | 0.449 | 0.002 | 0.42 | 1.470 | 0.359 | 24.39 |
| heptadecane | 0.614 | 0.014 | 2.35 | 0.003 | 0.000 | 11.80 |
| germacrene D | 0.642 | 0.014 | 2.10 | 0.014 | 0.002 | 12.84 |
| geranial | 1.100 | 0.000 | 0.03 | 2.869 | 0.455 | 15.85 |
| geranyl acetate | 0.744 | 0.013 | 1.71 | 0.235 | 0.014 | 6.06 |
| citronellol | 16.472 | 0.072 | 0.44 | 4.805 | 0.375 | 7.80 |
| 6-octen-1-ol, 7-methyl, 3-methylene | 0.242 | 0.010 | 4.13 | 0.115 | 0.013 | 11.20 |
| formic acid, 2-phenylethyl ester | 1.515 | 0.059 | 3.90 | 0.147 | 0.038 | 25.90 |
| geraniol | 2.798 | 0.060 | 2.14 | 9.320 | 0.873 | 9.37 |
| phenylethyl acetate | 1.779 | 0.012 | 0.67 | 0.363 | 0.062 | 17.01 |
| trans geraniol | 9.308 | 0.145 | 1.56 | 13.033 | 1.052 | 8.07 |
| benzyl alcohol | 2.857 | 0.010 | 0.35 | 9.850 | 1.675 | 17.00 |
| nonadecane | 0.560 | 0.057 | 10.10 | 0.010 | 0.005 | 47.37 |
| 2-phenyl ethyl alcohol (phenethyl alcohol) | 35.548 | 0.009 | 0.03 | 25.633 | 1.352 | 5.27 |
| eicosane | 0.016 | 0.000 | 1.71 | 0.053 | 0.015 | 28.38 |
| methyleugenol | 1.532 | 0.097 | 6.34 | 0.009 | 0.005 | 51.03 |
| heneicosane | 0.136 | 0.006 | 4.47 | 0.018 | 0.004 | 25.22 |
| eugenol | 4.033 | 0.220 | 5.46 | 0.054 | 0.013 | 24.22 |
| docosane | 0.004 | 0.000 | 5.36 | 0.032 | 0.005 | 16.70 |
| tricosane | 0.110 | 0.013 | 11.81 | 0.023 | 0.008 | 34.25 |

^a^ SD = standard deviation; CV%= coeffient of variation

**SUPPLEMENTARY FIGURES**

**Supplementary Fig. S1**

**
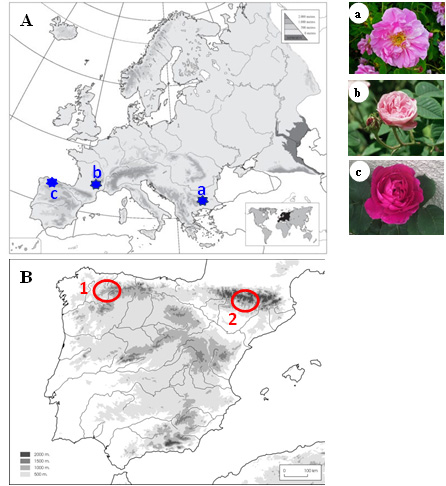
**

Supplementary Fig. S1: Cultivated and wild roses distribution. (A) Map of Europe showing current areas of perfume rose cultivation: (a) *R. damascena* in the Valley of Roses, Bulgaria, https://commons.wikimedia.org/wiki/File:Rosa_damascena_003.JPG H. Zell [CC BY-SA 3.0 (https://creativecommons.org/licenses/by-sa/3.0)]; (b) *R. centifolia* in Grasse, France, https://commons.wikimedia.org/wiki/File:Rosa_%27Petite_Lisette%27.jpg A. Barra [CC BY-SA 3.0 (http://creativecommons.org/licenses/by-sa/3.0/)] and (c) the examined material here named *Narcea*, in the Valle del Río Cibea (Asturias, Spain). (B) Map of Spain showing areas with abundant wild roses: (1) western Asturias, (2) the Aragonese Pyrenees.

**Supplementary Fig. S2**


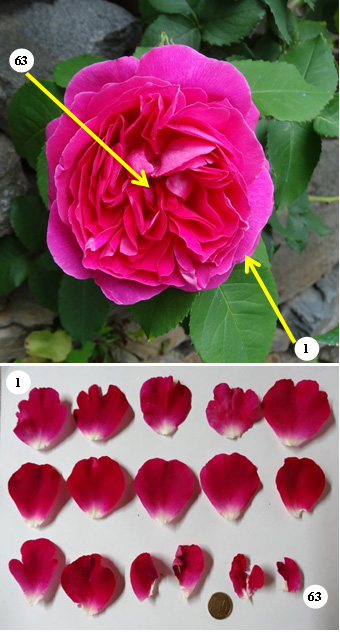


Supplementary Fig. S2Number and size of the different petals making a *Narcea rose* flower, and their position in the flower. Reference: 10 €cent coin.
